# Neuroanatomical Circuitry Associated with Exploratory Eye Movement in Schizophrenia: A Voxel-Based Morphometric Study

**DOI:** 10.1371/journal.pone.0025805

**Published:** 2011-10-03

**Authors:** Linlin Qiu, Lin Tian, Chao Pan, Risheng Zhu, Qi Liu, Jun Yan, Qiang Zhao, Huishu Yuan, Yonghua Han, Weihua Yue, Hao Yan, Dai Zhang

**Affiliations:** 1 Institute of Mental Health, Peking University, Beijing, People's Republic of China; 2 Key Laboratory for Mental Health, Ministry of Health, Beijing, People's Republic of China; 3 The Department of Radiology, The Third Hospital, Peking University, Beijing, People's Republic of China; The University of Hong Kong, Hong Kong

## Abstract

Schizophrenic patients present abnormalities in a variety of eye movement tasks. Exploratory eye movement (EEM) dysfunction appears to be particularly specific to schizophrenia. However, the underlying mechanisms of EEM dysfunction in schizophrenia are not clearly understood. To assess the potential neuroanatomical substrates of EEM, we recorded EEM performance and conducted a voxel-based morphometric analysis of gray matter in 33 schizophrenic patients and 29 well matched healthy controls. In schizophrenic patients, decreased responsive search score (RSS) and widespread gray matter density (GMD) reductions were observed. Moreover, the RSS was positively correlated with GMD in distributed brain regions in schizophrenic patients. Furthermore, in schizophrenic patients, some brain regions with neuroanatomical deficits overlapped with some ones associated with RSS. These brain regions constituted an occipito-tempro-frontal circuitry involved in visual information processing and eye movement control, including the left calcarine cortex [Brodmann area (BA) 17], the left cuneus (BA 18), the left superior occipital cortex (BA 18/19), the left superior frontal gyrus (BA 6), the left cerebellum, the right lingual cortex (BA 17/18), the right middle occipital cortex (BA19), the right inferior temporal cortex (BA 37), the right dorsolateral prefrontal cortex (BA 46) and bilateral precentral gyri (BA 6) extending to the frontal eye fields (FEF, BA 8). To our knowledge, we firstly reported empirical evidence that gray matter loss in the occipito-tempro-frontal neuroanatomical circuitry of visual processing system was associated with EEM performance in schizophrenia, which may be helpful for the future effort to reveal the underlying neural mechanisms for EEM disturbances in schizophrenia.

## Introduction

Eye movement abnormalities are among the most reproducible physiological dysfunctions associated with schizophrenia [1]–[3]. Particularly, exploratory eye movement (EEM) dysfunction appears to be specific to schizophrenia [4], [5]. EEM is a method to examine the participant's eye tracking while viewing stationary S-shaped figures. When performing the EEM task schizophrenic patients showed fewer eye fixations, longer mean duration of fixation and shorter mean scanning length than controls [6], [7]. In most previous EEM studies, the eye tracking patterns of schizophrenic patients were significantly different from those of normal individuals or patients with non-schizophrenic psychosis, and both the sensitivity and specificity of EEM were higher than 70% for discriminating schizophrenics from non-schizophrenics [4], [8]. Moreover, many studies have identified that the dysfunction patterns of EEM did not improve with relieved clinical symptoms of schizophrenia [6], [9]. Additionally, a 10-cM resolution genome-wide linkage analysis has suggested that the schizophrenia-related quantitative EEM trait was associated with chromosome 22q11.2 [10]. Thus, many investigators have proposed that EEM dysfunction appears to be a useful biological marker for schizophrenia [11]–[13].

The EEM task revealed the schizophrenia-related abnormalities by its 5 commonly used parameters obtained from the eye tracking data analysis, including number of eye fixations (NEF), total eye scanning length (TESL), mean eye scanning length (MESL), responsive search score (RSS) and cognitive search score (CSS). Numerous studies have found that the lower RSS was specific to schizophrenia [2], [12], [13]. RSS is obtained according to the pattern of eye fixation points after the final question, ‘Are there any other differences?’ The RSS abnormalities were only found in patients with schizophrenia [2], [12], [13], and the RSS of schizophrenic patients was significantly lower than that of depressed patients or healthy controls irrespective of geographical location, racial and culture influence [8]. Moreover, RSS impairments were also found in the healthy siblings of schizophrenic patients and therefore, it was thought to be an intermediate phenotype and a vulnerability marker for schizophrenia [14].

It is widely accepted that brain structural impairments and functional disability in schizophrenia could be attributed to its pathological substrates [15], [16]. Most studies investigating the neural substrate of eye movement have pointed that abnormal smooth pursuit and saccadic eye movements in schizophrenia were associated with specific brain structural abnormalities [17]–[19]. However, studies on exploring the underlying neuropathological mechanism of the EEM dysfunction in schizophrenia were relatively sparse. A pioneer study exploring the relationship between the EEM and brain morphology has presented that RSS was significantly related to gray matter density (GMD) in the right frontal eye field (rFEF) and right inferior frontal region in schizophrenia-spectrum patients [20]. The study increased the understanding of EEM abnormalities. However, the mechanism of EEM dysfunction in schizophrenia still needs to be further studied. We were wondering what the relationship between the EEM and brain morphology in healthy individuals is and whether or not the brain structural impairments in schizophrenic patients play a role in their EEM dysfunction.

In the current study, we conducted EEM tasks and high-resolution T1-weighted (MP-RAGE) anatomical magnetic resonance imaging (MRI) scans in schizophrenic patients and healthy controls to investigate the relationship between RSS and brain morphology by using a voxel-based morphometry (VBM) analysis.

## Methods

### Ethics statement

The study was approved by the Medical Research Ethics Committee of the Institute of Mental Health, Peking University. All participants enrolled in the study signed written informed consent.

### Participants

Thirty-three schizophrenic patients were recruited from the Institute of Mental Health, Peking University. All the patients satisfied ICD-10 diagnostic criteria for research for schizophrenia with paranoid subtype [21] and the diagnoses were made by two trained and experienced psychiatrists. Participants with epilepsy, mental retardation, severe physical disease, and treated with electroconvulsive therapy within the past 6 months were excluded. All patients received antipsychotic medications during the study. Antipsychotic dose was converted into chlorpromazine-equivalent dose [22]–[24]. The clinical symptoms of the patients were assessed by a trained and experienced psychiatrist with Positive and Negative Syndrome Scale (PANSS) [25]. Twenty-nine healthy controls, without a personal history of psychiatric illness or a family history of schizophrenia spectrum disorders, were recruited from community by advertisement. The schizophrenic patients and the healthy controls were matched for age, gender and years of education. All participants were right-handed and were screened to exclude neurologic disease, substance abuse, metal implant and a history of head injury. MRI scans and EEM tasks were conducted at the same day. Table 1 provided detailed demographic and clinical data.

**Table 1:** Demographic and clinical data of schizophrenic patients and healthy controls.

Participant characteristics	Schizophrenic patients (n = 33)	Healthy controls (n = 29)	p value
Male/female	19/14	17/12	0.934^a^
Age (years)	23.45 (4.05)^d^	23.17 (3.05)	0.107^b^
Education (years)	13.67 (2.12)	14.38 (1.97)	0.167^c^
Age at onset (years)	19.48 (3.37)	-	-
Duration of illness (months)	41.27 (33.09)	-	-
Drug (mg/day)^e^	437.27 (268.92)	-	-
PANSS^f^ positive score	19.64 (4.49)	-	-
PANSS negative score	15.94 (4.50)	-	-
PANSS total score	67.79 (11.47)	-	-

a Pearson Chi-square test.

b Two sample t-test.

c Mann-Whitney test.

d Mean (standard deviation).

e Chloropromazine-equivalent dose.

f Positive and Negative Syndrome Scale.

### Procedures

#### EEM data acquisition and processing

**Figure 1:**
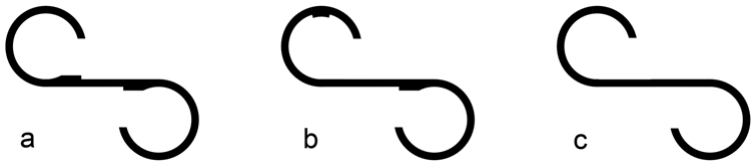
Figures in the exploratory eye movement task. (a) The original target figure. (b, c) Two figures that slightly differ from the target.

The recording procedure followed that used by Kojima et al. [2]. Each participant sat on a chair 1.5 m in front of a screen. A nac 8-B type Eye Mark Recorder (nac, Tokyo, Japan) and a device that detected corneal reflection of infrared light were equipped. Three stationary horizontal S-shaped figures were projected individually onto the screen, including one original target figure (Figure 1a) and two figures that slightly differed from the target (Figure 1b and Figure 1c). The width of each projected figure was 90 cm, and the height was 75 cm (angle of sight was 33° horizontally and 27.5° vertically). First, each participant was shown the original S-shaped figure (Figure 1a) for 15 seconds. Immediately after viewing it, the participant was asked to draw the target figure from memory (a retention task). Second, the participant was shown a slightly different figure with one bump in a different position (Figure 1b) for 15 seconds; Fifteen seconds later while the figure was still being viewed, the participant was asked if it differed from Figure 1a and, if so, how it differed. After the participant had replied, while still viewing the figure, he was then asked, ‘Are there any other differences?’ (This question was repeated until the participant stated there were no differences.) The second step (a comparison task) was repeated for an S-shaped figure without bumps (Figure 1c). Third, the participant was directed to view the projection of Figure 1a again for 15 seconds and to draw it again. The recordings of eye movements were stored into a video tape recording system and were analyzed with a computerized system.

**Figure 2:**
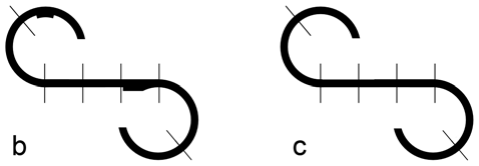
Figures for responsive search score calculation. Figure 1. b and c were each divided into seven sections.

The following 5 parameters were obtained:

1. During the first 15 seconds viewing of Figure 1a, number of eye fixations (NEF), total eye scanning length (TESL) and mean eye scanning length (MESL) were extracted. An eye fixation was defined as a gaze held for more than 200 milliseconds.
2. Responsive search score (RSS): Figure 1b and Figure 1c were each divided into seven sections (Figure 2). The participant was shown Figure 1b for 15 seconds and then was asked to tell the difference from Figure 1a and, if so, how it differed. The number of sections upon which the participant's eyes fixed at least once was counted in the 5 seconds immediately after the final question, ‘Are there any other differences?’ was asked. The procedure was repeated for Figure 1c. The possible total maximum score of RSS was 14.
3. Cognitive search score (CSS): When the participant viewed Figure 1b and Figure 1c for comparing to Figure 1a, the frequency of fixation points focused on the important areas on the figure was recorded. One score was got if the participant viewed any one of important areas of the two figures for three or more times within 15 seconds in the second step mentioned above. The possible total maximum score of CSS was 9. The measurements of RSS and CSS followed that conducted by Kojima et al. [12].

#### MRI data acquisition and processing

Structural MRI scans were obtained at the Department of the Radiology, the Third Hospital, Peking University, with a 3.0-Tesla Magnetom Trio MR system (Siemens Medical System, Erlangen, Germany). Three-dimensional T1-weighted images were acquired in a sagittal orientation employing a 3D-MPRAGE sequence with the following parameters: time repetition = 2350 ms, time echo = 3.44 ms, field of view = 256×256 mm^2^, flip angle = 7°, 192 sagittal slices, slice thickness = 1 mm, matrix size = 256×256, total acquisition time = 363 seconds. The MRI data were processed with the Statistical Parametric Mapping Software (SPM5, Welcome Department of Imaging Neuroscience, London; available at http://www.fil.ion.ucl.ac.uk/spm). A VBM analysis was performed using the VBM5 toolbox (available at http://dbm.neuro.uni-jena.de), which implements segmentation algorithm from SPM5 and extends the core segmentation algorithm by using the Hidden Markov Random Field (HMRF) approach [26]. A detailed description of processing procedure of the VBM5 toolbox can be found elsewhere [27]. This procedure yielded modulated and unmodulated types of tissues images. It has been suggested that in schizophrenic patients with cortical atrophy [28], modulation processing may not be suitable because it could have widespread effects on voxel signal intensities and may result in less consistent spatial localization of gray matter volume differences from sample to sample [29], [30]. Besides, the analysis of unmodulated data in schizophrenia is fertile [29], [31] and has been successfully conducted to explore the relationship between EEM and brain morphology in schizophrenia [20]. Thus we only used unmodulated data for GMD analysis in the present study. Resulting gray matter images were smoothed with a 12 mm full width-half maximum (FWHM) Gaussian kernel.

#### Statistical analysis

To test whether EEM were significantly altered in schizophrenic patients, comparisons of each EEM parameter between two groups was performed using t-test or the Mann-Whitney U-test as appropriate, given that MESL, CSS and RSS did not meet the criteria for normality (Shapiro-Wilk test). Pearson's correlation coefficient was used to identify significant relationships between each EEM parameter and duration of illness and medication respectively. Statistical significance was set at p<0.05 (two-tail). Statistical analysis was carried out with SPSS for windows (SPSS 13.0, SPSS Inc, Chicago, IL, USA).

The images were analyzed within the framework of the general linear model implemented in SPM5. First, to examine the GMD changes in schizophrenic patients, we used an analysis of covariance model with diagnostic group as fixed variable, including age and gender as confounding covariates. The significance level (p<0.05) corrected for multiple comparisons was estimated by combing a height threshold (p<0.001, two-tail, uncorrected) and a extent threshold at the cluster level [cluster extent (k)>620] using the random field theory [32]. Second, the relationship between GMD and RSS in schizophrenic patients and controls, respectively, was examined using a multiple regression model with RSS as independent variable. The multiple regression model for patients included antipsychotic dose, duration of illness, age and gender as confounding covariates; and the multiple regression model for controls included age and gender as confounding covariates. The significance level (p<0.05) corrected for multiple comparisons was estimated by combing a height threshold (p<0.01, one-tail, uncorrected) and a extent threshold at the cluster level (k>1683) using the random field theory [32]. Finally, to explore whether the deficits in gray matter was associated with the impaired RSS in schizophrenic patients, an overlap map was generated by calculating the intersection of the two thresholded statistical parameter maps. The results in the overlap mapping were saved to files and imported into the MarsBar toolbox (http://marsbar.sourceforge.net/) and the mean value of GMD of each region was calculated for each participant. Scatter plots of the relationships between RSS and the mean values of GMD were created using SPSS for windows.

## Results

### EEM results

Compared to healthy controls, schizophrenic patients showed significant decreases in RSS and CSS (see Table 2). There were no significant differences in the other three parameters of EEM (NEF, TESL and MESL) between groups. In addition, no eye movement parameters significantly correlated with antipsychotic dose and duration of illness respectively in schizophrenic patients (see Table S1).

**Table 2:** Comparison of eye movement parameters between schizophrenic patients and healthy controls.

Parameters	Schizophrenic patients (n = 33)	Healthy controls (n = 29)	t/z value	p value
NEF	23.21(6.49)^a^	24.28(5.78)	−0.678	0.501^b^
MESL	22.89(6.35)	28.13(11.81)	−1.877	0.061^c^
TESL	513.72(217.92)	610.59(180.87)	−1.889	0.064^b^
RSS	9.12(2.16)	10.52(1.43)	−2.807	0.005^c^
CSS	6.06(0.83)	6.72(0.70)	−3.282	0.001^c^

NEF, number of eye fixation; MESL, mean eye scanning length; TESL, total eye scanning length; RSS, responsive search score; CSS, cognitive search score.

a Mean (standard deviation).

b Two sample t-test.

c Mann-Whitney test.

### MRI Results

#### Morphology Analysis

**Figure 3:**
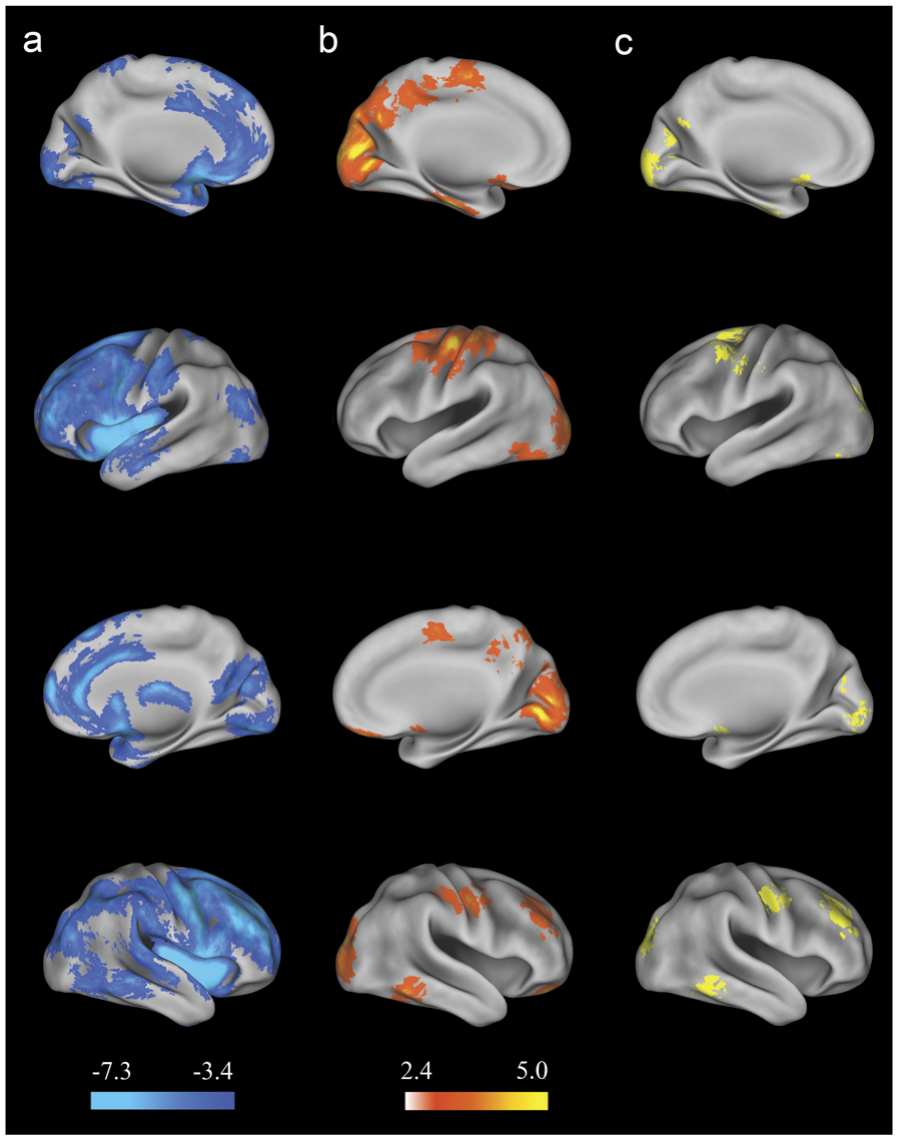
Surface renderings representing the results from the analysis of MRI data. Statistical parametric maps were superimposed on the population, landmarked-and surface-based (PALS) atlas of human cerebral cortex [73] using CARET software (http://brainvis.wustl.edu). Color bar indicates the t value. (a) Voxel-based morphometry analysis. Compared with healthy controls, regions with significantly (p<0.05, corrected) decreased gray matter density (GMD) in schizophrenic patients were shown in cool color. (b) Regression analysis of GMD with responsive search score (RSS) using antipsychotic dose, duration of illness, age and gender as confounding covariates in schizophrenic patients. Regions with significantly (p<0.05, corrected) positive correlations with RSS in schizophrenic patients were shown in warm color. (c) The result of overlap mapping was shown in yellow. Top row, left medial view; second row, left lateral view; third row, right medial view; fourth row, right lateral view.

Compared to healthy controls, schizophrenic patients showed significant GMD reductions in widespread brain areas such as the right middle frontal cortex, the right precuneus, the right cuneus, the right cerebellum, the left calcarine cortex, the left middle occipital cortex, the left superior temporal cortex, the left inferior temporal cortex, the left superior parietal cortex, the left paracentral lobule, bilateral lingual gyri and fusiform gyri (Figure 3a) (see Table S2). There were no significant GMD increases in schizophrenic patients compared to healthy controls.

#### Regression Analysis

In schizophrenic patients, RSS was positively correlated with GMD in the right middle frontal cortex, the right inferior temporal cortex, the right superior occipital cortex, the right cerebellum (lobule VII b), the left calcrine cortex, the left inferior occipital cortex, bilateral precentral gyri extending to the frontal eye fields (FEF, BA 8), cerebellum (crus I) and supplementary motor areas (Figure 3b) (see Table S3). No significantly positive correlation between RSS and GMD was found in healthy controls.

#### Overlap Mapping

**Figure 4:**
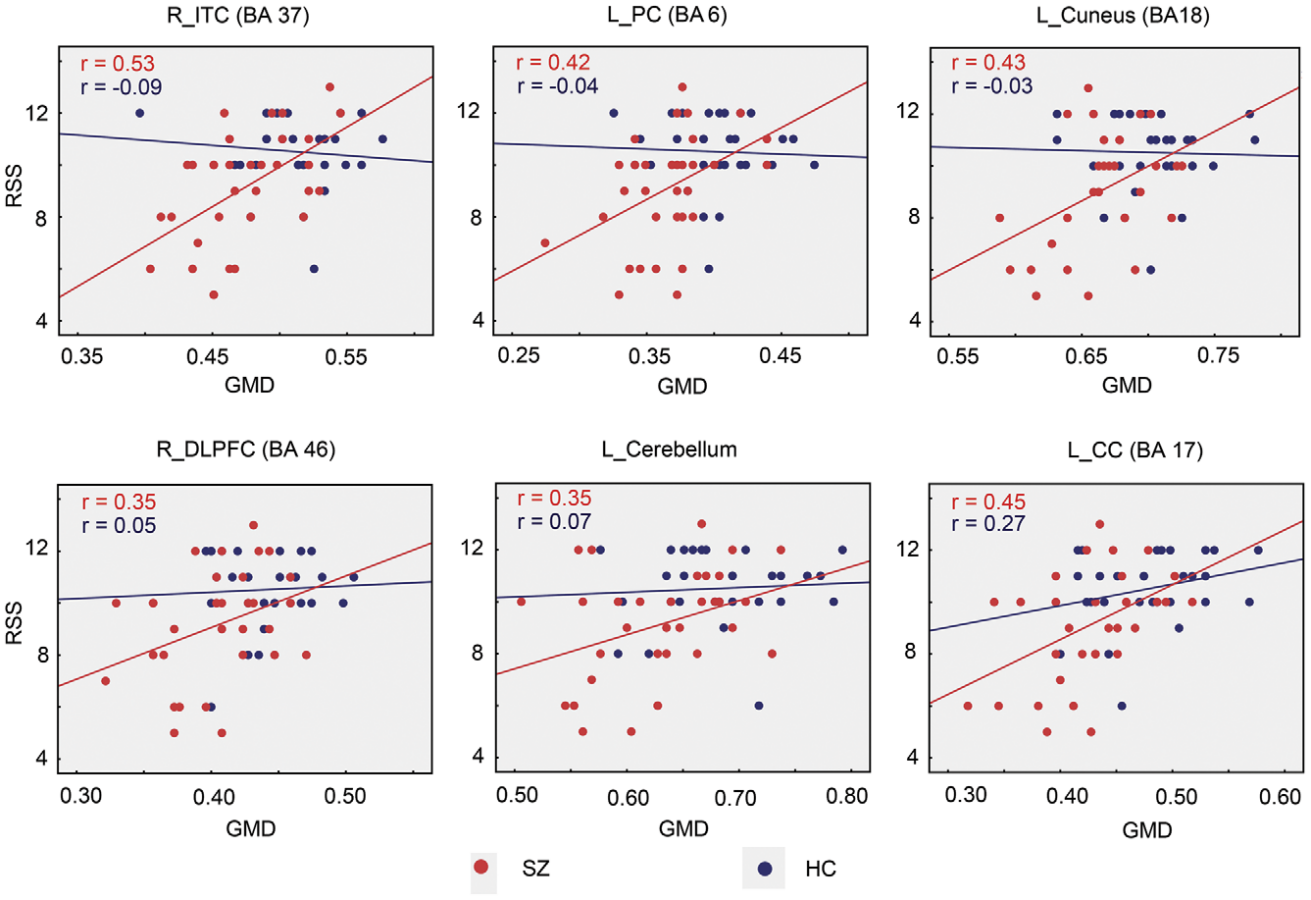
Representative scatter plots of the gray matter density (GMD) of overlap regions against responsive search score (RSS) in schizophrenic patients (SZ) and healthy controls (HC) with trend lines. Correlation coefficient (r) was used to assess the significance of a correlation (p<0.05). GMD values of the six overlap regions were significantly correlated with RSS in SZ, whereas there was no significant correlation between RSS and GMD in HC. Red lines for SZ and blue lines for HC. L, left; R, right; ITC, inferior temporal cortex; PC, precentral cortex; DLPFC, dorsolateral prefrontal cortex; CC, calcarine cortex.

In schizophrenic patients, overlap mapping revealed a number of brain regions not only with GMD reductions but also associated with RSS, such as the left calcarine cortex (BA 17), the left cuneus (BA 18), the left superior occipital cortex (BA 18/19), the left superior frontal gyrus (SFG, BA 6), the left cerebellum (crus I), the right lingual cortex (BA 17/18), the right middle occipital cortex (BA19), the right inferior temporal cortex (BA 37), the right dorsolateral prefrontal cortex (DLPFC) (BA 46) and bilateral precentral gyri (BA 6) extending to the FEF (BA8) (Figure 3c, Table 3). Scatter plots of the relationships between RSS and the mean values of GMD for several representative overlap regions were shown in Figure 4.

**Table 3:** Overlap mapping in schizophrenic patients.

Regions	Side	Brodmann areas	Cluster-size (k)^a^	Coordinates of geometric centers^b^
Middle frontal cortex	R	46	1828	39 43 34
Calcarine cortex	L	17	1761	−6 −100 −6
Precentral gyrus	R	6	932	50 −7 44
Superior frontal gyrus	L	6	791	−24 1 65
Middle occipital cortex	R	19	675	29 −89 24
Inferior temporal cortex	R	37	537	62 −51 −17
Cuneus	L	18	471	−1 −73 22
Superior occipital cortex	L	18/19	428	−26 −90 27
Fusiform cortex	L	20	412	−31 −17 −35
Precuneus	L	6	295	−44 2 55
Cerebellum	L	NA	152	−39 −76 −21
Lingual cortex	R	17/18	58	7 −90 −8
Postcentral cortex	L	4	55	−52 −10 45

a k = number of voxels in the particular cluster, only cluster-size (k) >10 voxels were listed.

b In Montreal Neurological Institute (MNI) space by using program fslstats implemented in FSL software (http://www.fmrib.ox.ac.uk/fsl/avwutils/index.html).

The brain imaging results were labeled with the Automated Anatomical Labeling (AAL) software [74] in combination with the Brodmann templates implemented in MRIcroN software (http://www.cabiatl.com/mricro/mricron). L, left; R, right.

## Discussion

The principal finding of this study was that lower RSS in schizophrenic patients was significantly associated with lower GMD in representative components of neural networks implicated in eye movement control and visual information processing, such as the bilateral BA 17, BA 18 and BA 19, bilateral precentral gyri (BA 6) extending to the FEF (BA 8), the right middle occipital cortex, inferior temporal cortex and DLPFC, the left SFG (BA 6) and left cerebellum (crus I). To our knowledge, we firstly reported empirical evidence that gray matter loss in the occipito-tempro-frontal neuroanatomical circuitry of visual processing system was associated with EEM performance in schizophrenia, which may be helpful for the future effort to reveal the underlying neural mechanisms for EEM disturbance in schizophrenia.

### Low RSS in schizophrenia

We found that schizophrenic patients showed lower RSS than healthy controls, in line with previous studies [2], [6], [8]. As an important parameter, RSS was based on the frequency of eye fixations on each section of a figure that occurred within 5 seconds immediately after the final question, ‘Are there any other differences?’ This search process could be accommodated within a visuooculomotor model [33]. First, a phase of perception (i.e., visual stimulus of the S-shaped figures), involving both the visual (occipital) and attentional (parietal) areas; Second, a phase of memorization, under the control of the cortical area actually involved in spatial working memory (corresponding to visuospatial integration); third, a phase of eye movement, under the control of the frontal and parietal motor areas. Therefore, the RSS was a measure of the ability for fine discrimination (i.e., figuring out the differences among three S-shaped figures), and, according to the abovementioned three phases, also reflected abilities to handle a series of processes, such as attention, perception, memory and execution. Thus, we speculated that the lower RSS at least partially resulted from the impairments of one or more of the abovementioned abilities in schizophrenia. Furthermore, obtaining RSS implicated a verbal communication during which ability of language comprehension was also needed. Therefore, RSS could also be an objective parameter for assessing the ability of personal communication, and decreased RSS might reflect the specific symptom of poor interpersonal communication in schizophrenics [2], [8]. In addition, consistent with previous reports [9], we also found that RSS showed no significant correlation with antipsychotic dose and duration of illness, which indicated that RSS was stable in schizophrenia and should be a trait-related deficit but not state-related deficit.

### Neuroanatomical circuitry underlying EEM

#### The ventral and dorsal visual-processing pathway

Our overlap mapping revealed that neuroanatomical deficits and EEM disturbances were associated in schizophrenic patients, i.e. some brain regions with gray matter loss were significantly positively correlated with RSS. Those brain regions included bilateral BA 17, BA 18, BA 19, and the right inferior temporal cortex. Most visual information arrives in the cortex via connections from the lateral geniculate nucleus to BA17 (also known as primary visual cortex, V1), from which two main pathways can be distinguished between areas of visual cortex: a dorsal (occipital-parietal) stream that reaches the posterior parietal cortex and a ventral (occipital-temporal) stream that goes to the inferior temporal region [34], [35]. Our current findings presented decreased GMD in BA17 (V1) and several areas involved in the ventral stream such as BA 18 (V2) and the inferior temporal cortex. Previous studies have reported that V1 is absolutely necessary for most visual functions, and lesions in V1 weakened the capacity for fine visual discrimination [36], [37]. Damage along the ventral visual stream was reported to result in several disturbances in visual recognition and understanding [38]. For instance, lesions in the inferior temporal cortex, which played a critical role in fine object recognition and discrimination tasks [39], [40], could lead to impairment in object shape recognition [41]. The overlap areas also included the right middle occipital cortices (BA19) and the left superior occipital cortex (BA 18/19), which were approximately corresponsive to V2 and V3 of the dorsal stream [35], [38], [42]. The dorsal stream has been documented with functions of visuomotor integration and spatial processing [43]. Furthermore, the middle occipital cortex was documented to associate with pursuit command, spatial position information and eye movement processing [44], [45]. O'Donnell et al. [46] reported that schizophrenia may be accompanied by impaired visual spatial perception and representation and suggested the deficits in trajectory discrimination might reflect a disturbance of the dorsal pathway of the visual system in schizophrenia. In fact, a growing body of evidence has documented deficits in perceptual discrimination of object form and spatial location in schizophrenic patients and, as our findings suggested, these perceptual deficits could be resulted from a relatively specific deficit in the ventral and dorsal visual-processing stream [46]–[49]. Besides, there are numerous interconnections, both forward and backward, between ventral and dorsal pathways, integrating the two pathways into a neural circuitry involved in processing visual information [38]. Thus, the disturbances in the neural circuitry of visual processing system may be associated with the EEM dysfunction in schizophrenia.

There was a concern whether decreased RSS was due to the decreased visual acuity in schizophrenic patients. It should be mentioned that each participant recruited in the present study could meet the basic visual demand of daily life and was able to complete the standard EEM task. Therefore, the changes of visual acuity could not account for decreased RSS in patients in the current study.

#### The frontal cortices

Our data clearly demonstrated that some prefrontal cortices, including the right DLPFC (BA 46) and the left SFG (BA 6), were brain areas with GMD reductions associated with RSS decreases. Eye movement dysfunctions in schizophrenia were reportedly concerned with higher-order cognition-related areas [50], [51], among which the prefrontal cortices are likely involved in task-dependent interpretation of visual input to orchestrate cognitive decisions [52]. For instance, Bagary et al. [17] demonstrated that oculomotor dysfunction in schizophrenic patients correlated with abnormal gray matter in DLPFC. Courtney et al. [53] suggested that the SFG was specialized for spatial working memory, which was found to be associated with impaired oculomotor task performance in schizophrenic patients [54]. In addition, a transcranial magnetic stimulation study also showed that the SFG was involved in visual-spatial working memory task, whereas the DLPFC interfered with both visual-object and visual-spatial information in humans [55]. As aforementioned, the procedure of obtaining RSS implicated attention, working memory, as well as eye movement control, during which the DLPFC and SFG may be recruited. Thus, gray matter loss in the right DLPFC and the left SFG may be implicated with the dysfunction of EEM in schizophrenic patients.

The bilateral precentral gyri (BA6) extending to the FEF (BA 8) was another finding in our overlap mapping analysis. The FEF was a principal region involved in oculomotor control and maintenance of eye fixation [56], [57]. Many lesion and functional neuroimaging studies emphasized that the FEF was concerned primarily with the visual attention, the actual triggering and execution of eye movements in human [58], [59]. The FEF lesions have been found to impair the oculomotor tasks requiring exploration or orientation towards visual stimuli [60]. In addition, the FEF may be particularly important in a series of eye movement tasks like smooth pursuit eye movement, saccade eye movement and EEM in schizophrenia [20], [50], [51]. Tsunoda et al. [20] found that the RSS was positively correlated with GMD in the FEF in schizophrenia spectrum patients and suggested that the FEF-related neural network dysfunction may be associated with the systematic intentional exploration of space and contribute to the EEM disturbance in patients. In this study, we replicated their findings on the relationship between the FEF impairment and EEM dysfunction in schizophrenic patients.

Taken together, the cerebral cortical areas observed in our overlap mapping were compatible with a recent study that schizophrenia showed severe functional deficits across a distributed network of sensory and higher-order cognitive brain regions in visual object processing [61]. Although the causal relationship between gray mater changes and EEM abnormalities is still unclear, according to our findings, we speculated that neuroanatomical deficits in the dorsal and ventral pathways, and ‘high-order’ frontal areas might induce a dysintegration of the occipito-tempro-frontal circuitry and dysfunction within this circuitry may underlie the disturbances of EEM in schizophrenia.

#### The cerebellum

Our findings also showed the GMD of cerebellum (crusI, lobule VII b) was associated with EEM in schizophrenic patients. The cerebellum is commonly regarded as an organ that subserves coordination, balance, and fine motor control [62]. Moreover, its cortex and deep cerebellar nuclei play a crucial role in supporting the accuracy and adaptation of voluntary eye movements [63]. Previous studies gave evidence that multiple cerebellar structures are involved in the control of eye movements, among which the oculomotor vermis (lobuli VI and VII) are especially well documented [63]. The vermis lesions led to the disruption of the timing, accuracy and dynamics of saccades, and the ability to maintain eye fixation as well [64], [65]. Furthermore, neural cells located in the crus I and II were recorded to discharge related to eye movements and visual stimuli in monkeys during a visually guided eye movement tracking task [66]. In human, lesions of the lateral cerebellar hemisphere may affect the smooth pursuit eye movement [67]. In addition, lateral cerebellar hemisphere (particularly in the crus I and II) involved in human executive network and played an important role in executive functions, such as reasoning and working memory, which were indispensable in EEM as well [68], [69]. Hence, besides the cerebral neuroanatomical circuitry of visual processing system aforementioned, the cerebellum impairment may be also implicated with the EEM dysfunction in schizophrenic patients.

#### Findings in healthy controls

Another goal of the present case-control study was to explore the relationship pattern between RSS and GMD in healthy individuals. We found no significantly positive correlation between RSS and GMD in controls, which may be due to the relatively centralized RSS as showed from the lower standard deviation in controls. Previous studies have suggested that multiple cortical areas such as frontal eye fields, the middle temporal, brain stem and cerebellum are all human eye-movement-related structures [63], [70]. Moreover, a PET study indicated a network of brain regions including frontal eye fields, supplementary eye field, V5 and medial cuneus was activated during smooth pursuit and saccade eye movements in healthy individuals [71]. However, the neurobiological pathways implicating EEM in healthy individuals is still unclear. It is required to integrate multiple research techniques, e.g. functional MRI and PET, to address this question in the future studies.

### Limitations and considerations

The present study has several limitations and considerations. First, patients in our study had relatively discrete durations of illness (from 8.18 to 74.36 months) and were all exposed to antipsychotic medication. The exposure to antipsychotic medication may impact brain structure [72], which was reflected in our data as well (see Figure S1, Table S4). Although we included antipsychotic dose and duration of illness as nuisance covariates in the regression model for schizophrenic patients, their underlying effects on brain structure cannot be removed thoroughly. Second, the RSS has been found to be correlated positively with the performance Intelligence Quotient (IQ) and the Wechsler's Maze test in schizophrenic patients [2]. However, we did not include IQ or other measurements of cognitive function as control factors in the current study. Though without IQ, a measure of general cognitive function, all participants in the current study were at least junior high school graduates and the education levels were well matched between groups. In addition, patients with mental retardation were excluded from our study. Therefore, we inferred that the intelligence level of all the participants might be normal. Now the effects of the cognitive function on the EEM performance were still unclear. Thus, it will be helpful to elucidate the relationship between the cognitive function and EEM by collecting data from multiple dimensions of cognition and using multiple regression analysis to examine the relationship between the cognitive measurements and EEM performance in future study. Additionally, schizophrenia is a highly genetic disease and EEM is an intermediate phenotype of schizophrenia. Thus, it would be of particular interest to consider the relationship between EEM and brain structure in unaffected relatives of schizophrenic patients in future work.

### Conclusions

In summary, decreased RSS and widespread gray matter loss were observed in schizophrenic patients. Moreover, we found that decreased RSS in schizophrenic patients was correlated with decreased GMD in several brain areas in a distributed occipito-tempro-frontal circuitry involved in visual information processing and eye movement control. These findings revealed an association between gray matter reductions and EEM abnormalities, which may help to reveal the underlying neural mechanisms for EEM disturbances in schizophrenia in the future study. The present work may well provide a new underpin for the further research to evaluate and verify the EEM as a biological marker for schizophrenia.

## Supporting Information

Figure S1 **Surface renderings representing the results from the analysis of MRI data.** Statistical parametric maps of regression analysis in schizophrenic patients was superimposed on the population, landmarked-and surface-based (PALS) atlas of human cerebral cortex [73] using CARET software (http://brainvis.wustl.edu). In the regression analysis, chlorpromazine-equivalent antipsychotic dose was used as independent variable and gray matter density within each voxel as dependent variable, including age and gender as confounding covariates. Regions with significantly (p<0.01, corrected) negative correlations with RSS in schizophrenic patients were shown in cool color. Color bar indicates the t value.
(TIF)

Table S1 **The relationship between parameters of EEM and antipsychotic dose and duration of illness in schizophrenic patients.**
(DOC)

Table S2 **Brain regions with decreased gray matter density in schizophrenic patients.**
(DOC)

Table S3 **Brain regions with a significant positive correlation between responsive search score and gray matter density in schizophrenic patients.**
(DOC)

Table S4 **Brain regions with a significant negative correlation between antipsychotic medication and gray matter density in schizophrenic patients.**
(DOC)
