# Supplementary figures and images for: Neuroanatomical Circuitry Associated with Exploratory Eye Movement in Schizophrenia: A Voxel-Based Morphometric Study
Source: PLoS One. 2011 Oct 3;6(10):e25805. doi: 10.1371/journal.pone.0025805 (PMC3185013; doi:10.1371/journal.pone.0025805)

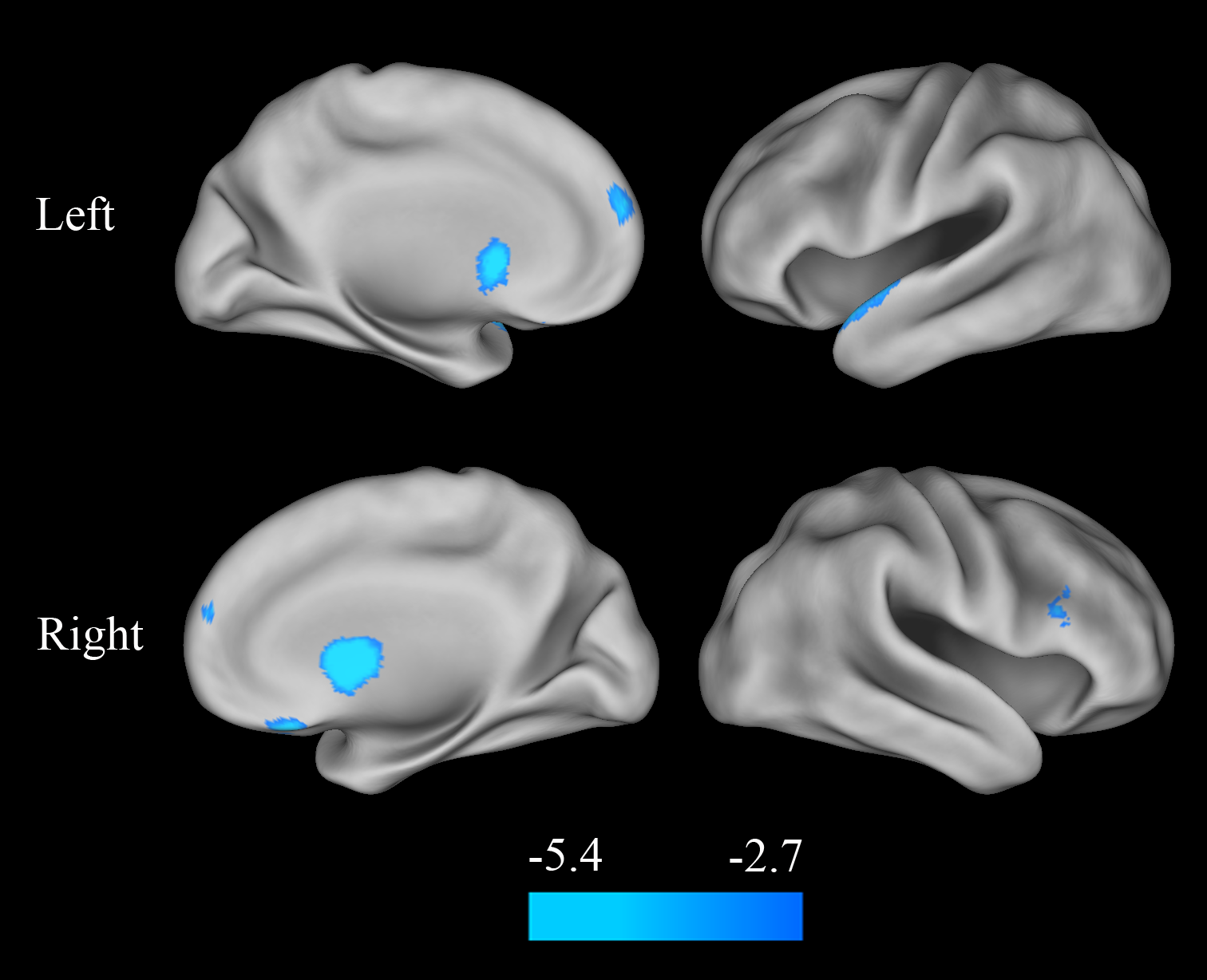

Supplement: Figure S1 — Surface renderings representing the results from the analysis of MRI data. Statistical parametric maps of regression analysis in schizophrenic patients was superimposed on the population, landmarked-and surface-based (PALS) atlas of human cerebral cortex [73] using CARET software (http://brainvis.wustl.edu). In the regression analysis, chlorpromazine-equivalent antipsychotic dose was used as independent variable and gray matter density within each voxel as dependent variable, including age and gender as confounding covariates. Regions with significantly (p<0.01, corrected) negative correlations with RSS in schizophrenic patients were shown in cool color. Color bar indicates the t value. (TIF) [file pone.0025805.s001.tif]
