# Supplementary material for: Neuroanatomical Circuitry Associated with Exploratory Eye Movement in Schizophrenia: A Voxel-Based Morphometric Study
Source: PLoS One. 2011 Oct 3;6(10):e25805. doi: 10.1371/journal.pone.0025805 (PMC3185013; doi:10.1371/journal.pone.0025805)
Supplement: Table S1 — The relationship between parameters of EEM and antipsychotic dose and duration of illness in schizophrenic patients. (DOC) [file pone.0025805.s002.doc]

**Table S1. The relationship between parameters of exploratory eye movement and antipsychotic dose and duration of illness in schizophrenic patients.**

|  | **NEF** | **MESL** | **TESL** | **CSS** | **RSS** |
| --- | --- | --- | --- | --- | --- |
| **Antipsychotic dose** | -0.278^▼^ | 0.336 | 0.013 | -0.135 | 0.238 |
| **Duration of illness** | 0.094 | 0.341 | 0.302 | 0.206 | -0.114 |

^▼^Correlation coefficients.

Pearson’s correlation was used since the clinical variables and all the exploratory eye movement (EEM) parameters met the criteria for normality (Shapiro-Wilk test). There was no significant correlation between clinical variables (antipsychotic dose and duration of illness) and parameters of EEM at the significant level of p < 0.05 (2-tailed). NEF, number of eye fixation; MESL, mean eye scanning length; TESL, total eye scanning length; RSS, responsive search score; CSS, cognitive search score.
