# Supplementary material for: Neuroanatomical Circuitry Associated with Exploratory Eye Movement in Schizophrenia: A Voxel-Based Morphometric Study
Source: PLoS One. 2011 Oct 3;6(10):e25805. doi: 10.1371/journal.pone.0025805 (PMC3185013; doi:10.1371/journal.pone.0025805)
Supplement: Table S2 — Brain regions with decreased gray matter density in schizophrenic patients. (DOC) [file pone.0025805.s003.doc]

**Table S2**. Brain regions with decreased gray matter density in schizophrenic patients.

| **Regions** | **Cluster-size (k)** | **t-scores of peak voxel** | **Coordinates of peak voxel in MNI space** |
| --- | --- | --- | --- |
| Frontal_Middle_R | 182096 | 7.30 | 45 38 20 |
| Frontal_Middle_R |  | 6.99 | 39 5 55 |
| Temporal_Superior_L |  | 6.86 | -57 0 -2 |
| Fusiform_R | 6948 | 5.65 | 26 -76 -13 |
| Lingual_R |  | 5.11 | 16 -68 -11 |
| Lingual_R |  | 5.09 | 12 -84 -14 |
| Parietal_Superior_L | 1729 | 5.44 | -19 -50 72 |
| Paracentral_Lobule_L |  | 3.73 | -12 -35 75 |
| Cuneus_R | 5081 | 5.18 | 14 -72 31 |
| Precuneus_R |  | 4.89 | 18 -67 26 |
| Precuneus_R |  | 4.89 | 5 -71 22 |
| Fusiform_R | 1930 | 5.06 | 36 -7 -43 |
| Lingual_L | 4725 | 5.03 | -15 -99 -17 |
| Fusiform_L |  | 4.97 | -33 -77 -18 |
| Calcarine_L |  | 4.74 | -10 -103 -10 |
| Occipital_Middle_L | 831 | 4.74 | -28 -89 29 |
| Cerebelum_9_R | 3848 | 3.85 | 5 -46 -44 |
| Occipital_Middle_L | 980 | 4.65 | -46 -76 23 |
| Temporal_Inferior_L | 3899 | 4.59 | -46 -28 -25 |
| Fusiform_L |  | 4.53 | -37 -18 -35 |
| Temporal_Inferior_L |  | 4.32 | -31 -6 -42 |
| Cerebelum_8_L | 1014 | 4.28 | -15 -70 -53 |
| Temporal_Inferior_L | 725 | 4.24 | -58 -42 -20 |

The brain imaging results reported were labeled with the Automated Anatomical Labeling (AAL) software [1]. Anatomical labels of peak coordinates were reported in Montreal Neurological Institute (MNI) space. L = left; R = right; k = number of voxels in the particular cluster.

**References**

1. Tzourio-Mazoyer N, Landeau B, Papathanassiou D, Crivello F, Etard O, et al. (2002) Automated anatomical labeling of activations in SPM using a macroscopic anatomical parcellation of the MNI MRI single-subject brain. Neuroimage 15: 273-289.
