# Supplementary material for: Neuroanatomical Circuitry Associated with Exploratory Eye Movement in Schizophrenia: A Voxel-Based Morphometric Study
Source: PLoS One. 2011 Oct 3;6(10):e25805. doi: 10.1371/journal.pone.0025805 (PMC3185013; doi:10.1371/journal.pone.0025805)
Supplement: Table S3 — Brain regions with a significant positive correlation between responsive search score and gray matter density in schizophrenic patients. (DOC) [file pone.0025805.s004.doc]

**Table S3. Brain regions with a significant positive correlation between responsive search score and gray matter density in schizophrenic patients.**

| **Regions** | **Cluster-size (k)** | **t-scores of peak voxel** | **Coordinates of peak voxel in MNI space** |
| --- | --- | --- | --- |
| Precentral_L | 9246 | 4.95 | -41 -17 60 |
| Precentral_L |  | 3.49 | -42 0 58 |
| Temporal_Inferior_R | 5250 | 4.75 | 64 -51 -20 |
| Cerebelum_Crus1_R |  | 3.52 | 47 -54 -33 |
| Cerebelum_VII b_R |  | 3.19 | 42 -50 -48 |
| Occipital_Superior_R | 35061 | 4.41 | 24 -92 22 |
| Calcarine_L |  | 4.39 | -1 -87 0 |
| Frontal_Middle_R | 2709 | 4.23 | 36 37 46 |
| Frontal_Middle_R |  | 3.70 | 44 46 32 |
| Frontal_Middle_R |  | 3.47 | 37 43 35 |
| Precentral_R | 2406 | 4.12 | 50 -5 43 |
| Postcentral_R |  | 3.35 | 49 -12 39 |
| Postcentral_R |  | 3.05 | 47 -21 45 |
| Frontal_Middle_Orbital_R |  | 2.57 | 24 50 -19 |
| Cerebelum_Crust_L | 3764 | 3.83 | -43 -73 -29 |
| Occipital_inferior_L |  | 2.67 | -47 -69 -20 |
| Supplementary_Motor_Area_L | 2962 | 3.77 | -9 -10 63 |
| Supplementary_Motor_Area_R |  | 3.53 | 7 -10 54 |
| Supplementary_Motor_Area_L |  | 3.52 | -19 -3 65 |
| Fusiform_L |  | 3.44 | -26 -15 -34 |
| Frontal_Superior_Orbital_L | 1780 | 3.55 | -14 13 -21 |

The brain imaging results reported were labeled with the Automated Anatomical Labeling (AAL) software [1]. Anatomical labels of peak coordinates were reported in Montreal Neurological Institute (MNI) space. L = left; R = right; k = number of voxels in the particular cluster.

**References**

1. Tzourio-Mazoyer N, Landeau B, Papathanassiou D, Crivello F, Etard O, et al. (2002) Automated anatomical labeling of activations in SPM using a macroscopic anatomical parcellation of the MNI MRI single-subject brain. Neuroimage 15: 273-289.
