# Supplementary material for: Neuroanatomical Circuitry Associated with Exploratory Eye Movement in Schizophrenia: A Voxel-Based Morphometric Study
Source: PLoS One. 2011 Oct 3;6(10):e25805. doi: 10.1371/journal.pone.0025805 (PMC3185013; doi:10.1371/journal.pone.0025805)
Supplement: Table S4 — Brain regions with a significant negative correlation between antipsychotic medication and gray matter density in schizophrenic patients. (DOC) [file pone.0025805.s005.doc]

**Table S4. Brain regions with a significant negative correlation between antipsychotic medication and gray matter density in schizophrenic patients.**

| **Regions** | **Cluster-size (k)** | **t-scores of peak voxel** | **Coordinates of peak voxel in MNI space** |
| --- | --- | --- | --- |
| Rectus_R | 2250 | 5.35 | 5 30 -34 |
| Frontal_Inferior_R | 1170 | 4.76 | 58 23 24 |
| Temporal_Pole_superior_L | 2436 | 3.85 | -51 13 -14 |
| Temporal_Superior_L |  | 3.48 | -59 0 -3 |
| Temporal_Superior_L |  | 3.43 | -60 5 -13 |
| Frontal_Superior_Medial_L | 1582 | 3.69 | -4 61 21 |

The brain imaging results reported were labeled with the Automated Anatomical Labeling (AAL) software [1]. Anatomical labels of peak coordinates were reported in Montreal Neurological Institute (MNI) space. L = left; R = right; k = number of voxels in the particular cluster.

**References**

1. Tzourio-Mazoyer N, Landeau B, Papathanassiou D, Crivello F, Etard O, et al. (2002) Automated anatomical labeling of activations in SPM using a macroscopic anatomical parcellation of the MNI MRI single-subject brain. Neuroimage 15: 273-289.
